# Progress in Diseases of the Blood

**Published:** 1904-03-05

**Authors:** 


### PROGRESS IN DISEASES OF THE BLOOD.

(*Concluded from page* 393.)

**Leukæmia.**—An acute case of the *myelogenous form* is reported by Billings and Capps,[7] who collected records of seven instances which all began suddenly. Hæmorrhages, fever, severe anæmia, with white cells varying from 16,000 to 540,000, and largely composed of myelocytes and large mononuclear ones, were present in all. The cervical glands were usually enlarged. Parkes Weber, at the Pathological Society, described a case of the *lymphatic type*, and thinks that these acute cases are of a less differentiated type than the chronic ones. A. O. Kelly, at the American Association, brought forward four more of a typical lymphatic type. There were no myelocytes or granular cells in the bone marrow, and the writer notes that the disease may occur without enlargement of the glands. Kinnicutt reported another case at the same time, and also referred to the absence of myelocytes in the marrow. The spleen and liver were enlarged. Cabot doubted whether lymphatic leukæmia was a definite disease, as he had seen similar blood states in cases of sepsis, which rapidly recovered. Middleton[8] gives an account of a Glasgow case, which died three months after the first glandular enlargement under the chin was noticed. A month before death there was very slight increase in the white cells, but the blood became typical later on. Ewing has remarked on the fewness of the leucocytes in some acute cases. Lindsay Stevens, at the Glasgow Med. Chirurgical, reported one where subcutaneous nodules were present, resembling those in melanotic sarcoma. As in the last case, there was a huge percentage of large lymphocytes. An instance of the chronic type is given by O. K. Williamson,[9] where one feature of the acute form was present, viz., a large number of nucleated red cells, but the duration and the numerous small lymphocytes, and the absence of poikilocytosis pointed the other way. Barie and Salmon, of Paris, at the Société Médicale, gave an account of a patient who died after six weeks' illness, with 1,000,000 leucocytes and fatal hæmorrhage from a subcutaneous hæmatoma. Thus 10 new cases of acute leukæmia have been reported in a year.

**Worm Infections.**—A. E. Boycott[10] writes of the blood-counts found when parasitic worms are present. In 19 cases of thread-worms less than half showed any excess of eosinophile cells, and other evidence is given to show that many wormless children have this excess. In nine cases of tænia, ascaris and bilharzia, the eosinophiles were most irregular, in some cases they were normal, and in some increased up to 47·6 per cent. Hence the test is an uncertain one. Recently, interest has arisen from the outbreak of ankylostomiasis in Cornwall, and the danger of it spreading to other English mines. R. Stockman[11] has found a case in Scotland in a coal-miner who had been anæmic since his service in India. The hæmoglobin was only 28 per cent., and the eosin cells were in excess, while ova of the parasite were found in the fæces. Under 20-grain doses of thymol, preceded and followed some hours after by castor oil, he made a complete recovery. Three doses in all were given. Cases left untreated may end fatally from the great loss of blood, but if the worms are few, and no fresh infection occurs, the parasites die in about six years. At the International Miners' Congress it was stated that 25,000 miners in one coalfield alone were affected. At Liège 75 to 80 per cent. of the miners were infected. It was promptly stamped out in France when it first appeared, but the German and Belgian authorities had failed to combat the disease in those countries.

**Lymph in the Tissues.**—G. Oliver[12] takes the difference in the blood-count, before and after tight rubber rings have been rolled up the fingers, as a measure of the tissue lymph, and as a means of studying its circulation. Thus he lays down that after food there is a rapid flow of this lymph into the tissue spaces, and this is followed by a rise in the corpuscles and hæmoglobin of the blood. There is a rhythmical transfer of fluid between the blood-vessels and the tissues, amounting to about 28 ozs. in an average man after a meal, from the vessels into the lymph spaces, so that we get therefore an immediate flow of nourishment into the tissues after a meal long before the food itself has been digested and absorbed, a theory which is supported by our ordinary sensations. In cases of high tension there is a failure in the return flow, that is, in emptying the lymph spaces. The pathology of gout is, he thinks, to be largely explained by the failure of this "intermediary circulation."

[7] Med. Rec., Sept. 19. [8] Lancet, July 18. [9] Brit. Med. Jour Nov. 14. [10] Ibid., Nov. 14. [11] Brit. Med. Jour., July 25. [12] Lancet, Oct. 3.

